# A rare combination of tibial artery aneurysm and traumatic arteriovenous fistula: a case report

**DOI:** 10.11604/pamj.2022.42.122.29735

**Published:** 2022-06-15

**Authors:** Chrysanthi Papageorgopoulou, Konstantinos Nikolakopoulos, Ioannis Ntouvas, Spyros Papadoulas, Neoklis Kritikos

**Affiliations:** 1 Department of Vascular Surgery, University General Hospital of Patras, Patras, Greece,; 2 Vascular Unit, Department of Surgery, General Hospital of Patras, Patras, Greece,; 3 Department of Surgery, General Hospital of Kalamata, Kalamata, Greece

**Keywords:** Tibial artery aneurysm, endovascular treatment, arteriovenous fistula, trauma, case report

## Abstract

Aneurysms of the tibial arteries are rare, and they most commonly occur due to trauma of the lower extremities. Acquired arteriovenous fistulas of the tibial arteries are a recognized complication of trauma of the lower extremity and they are related to the mechanism of the injury. Although the natural history of stable arteriovenous fistula is relatively benign, the aneurysms of the tibial arteries should be repaired with autologous vein bypass grafts, if there is distal ischemia. Endovascular repair has been reported as an effective alternative treatment for traumatic tibial artery aneurysms. The case of a 60-year-old male with the rare combination of a tibial artery aneurysm and traumatic arteriovenous fistula of the left lower extremity is reported in view of its rarity and the modern holistic endovascular approach used for treatment. A covered stent graft in the anterior tibial artery was used for the treatment of both the aneurysm and the arteriovenous fistula. The patient was discharged under dual antiplatelet treatment for three months, continued by clopidogrel as monotherapy. At the six-month follow-up, the patient has restored palpable peripheral pulses and no sign of recurrence or complication was recognized. A total endovascular approach can be an effective treatment for these complicated traumatic aneurysms.

## Introduction

Aneurysms of the tibial arteries are rare entities, and only case reports have been reported to published literature [1,2]. The most common cause of this rare type of aneurysm is the trauma, including the penetrating trauma and fractures, but also iatrogenic injuries [3,4] have been reported as an important cause of tibial artery aneurysms. Arteriovenous Fistulas (AVFs) can be either congenital or acquired. Acquired AVFs, in the civilian population, involve the upper and lower extremities in 22% and 20% of cases, respectively. The main cause of acquired arteriovenous fistulas remain the trauma, but iatrogenic injuries from percutaneous cardiac and vascular procedures, central venous catheterization, orthopedic procedures on the lumbar spine and knee, diagnostic biopsies and percutaneous insertion of drainage catheters can result to traumatic arteriovenous fistulas. The most common site of iatrogenic injury in the lower extremity is the right common femoral artery [5]. Although the natural history of stable arteriovenous fistula is known and relatively benign, the aneurysms of the tibial arteries should be repaired, if there is distal ischemia. Endovascular repair has been reported in case reports, as an effective alternative treatment of tibial artery aneurysms. With this case report, we present the rare combination of a tibial artery aneurysm and a traumatic arteriovenous fistula in the lower extremity, treated only via endovascular repair.

## Patient and observation

**Patient information:** a 55-year-old male was admitted to the emergency department of our hospital due to oedema on the anterior surface of the tibia. The patient had a history of a superficial trauma on the anterior-lateral surface of the tibia two days ago, during working on a metallic object with a hammer. No family medical history of aneurysmatic disease was reported.

**Clinical findings:** the physical examination revealed mild oedema above the area of the tibia, a small traumatic lesion of the skin of the tibia and a pulsatile mass with an audible bruit over the anterior surface of the tibia.

**Figure 1:**
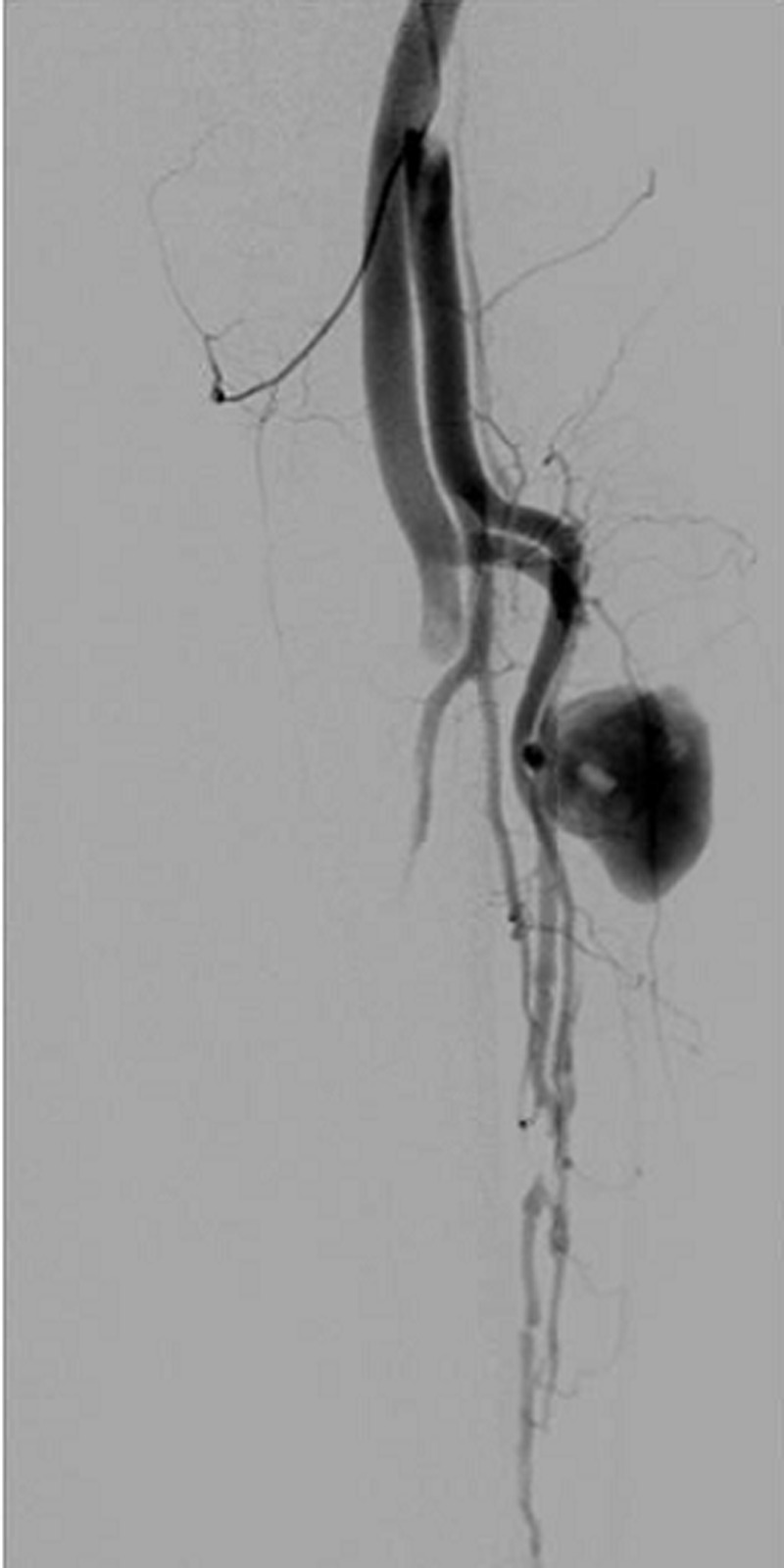
a digital subtraction angiography was performed and the diagnosis of the anterior tibial artery aneurysm was confirmed

**Diagnostic assessment:** the X-rays of the lower extremity revealed a small metal fragment on the tibiofibular clear space. Duplex ultrasound scanning was performed and a false aneurysm of the anterior tibial artery with a maximum transverse diameter of 5cm, with mural thrombus and a traumatic arteriovenous fistula were diagnosed. A digital subtraction angiography was performed confirming the diagnosis (Figure 1).

**Figure 2:**
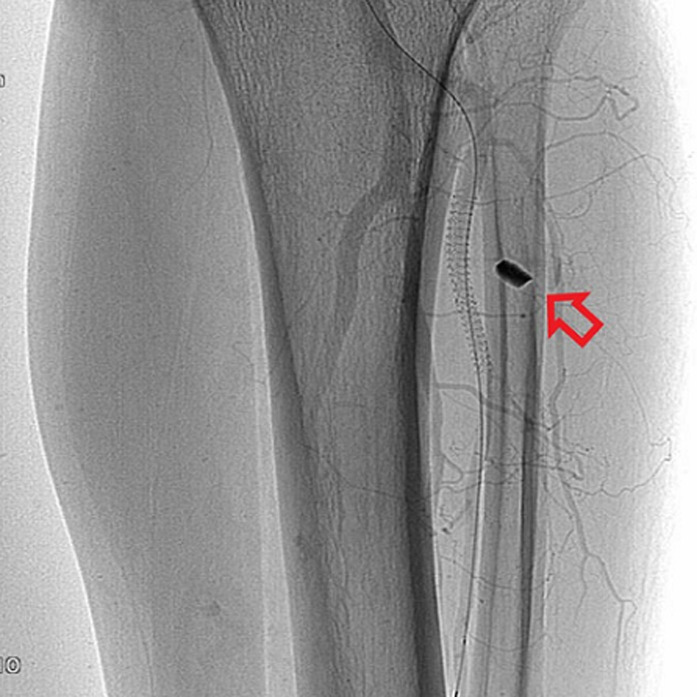
a covered stent was used as treat the anterior tibial artery aneurysm and the traumatic fistula, the metal fragment is easily recognized (arrow)

**Figure 3:**
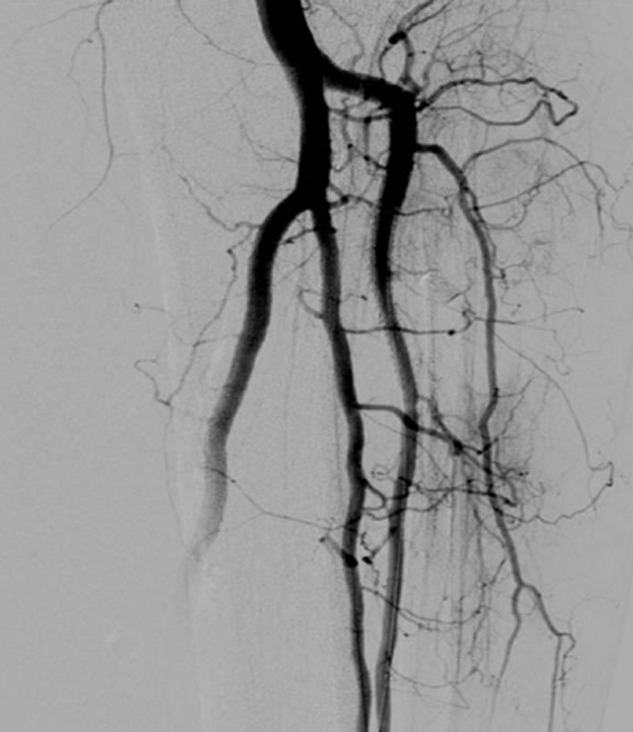
after the deployment of the stent graft, the blood flow through the anterior tibial artery was restored

**Therapeutic interventions:** at the same time with the digital subtraction angiography, a covered stent graft was deployed in the anterior tibial artery (Figure 2) to treat both the aneurysm and the arteriovenous fistula (Figure 3).

**Follow-up and outcomes:** the patient was discharged at the third postoperative day under dual antiplatelet treatment with clopidogrel 75 mg and acetylsalicylic acid 100 mg once daily for three months, continued by clopidogrel 75 mg once daily, as monotherapy. At the six-month follow-up, the patient has restored palpable peripheral pulses and no sign of recurrence or complication was recognized.

## Discussion

Aneurysms of the tibial arteries are a rare entity and only case reports have been reported to published literature [1,2]. The most common causes of this rare type of aneurysm are the penetrating trauma, fractures and iatrogenic injury during orthopedic surgeries [3] or peripheral endovascular interventions, including trauma from catheterization or thromboembolectomy balloon catheters [4]. Our patient had a symptomatic traumatic aneurysm due to a metal fragment and according to literature these should be repaired with autologous vein bypass grafts if there is distal ischemia [1,2]. If all tibial arteries are patent before the treatment of the aneurysm, proximal and distal ligation of the aneurysm can be the proper way of treatment. Although, many endovascular techniques have been used for other peripheral artery aneurysms, only coil embolization [6] has been reported as an effective treatment for tibial artery aneurysms. Arteriovenous fistulas (AVFs) can be either congenital or acquired. The main cause of acquired arteriovenous fistulas remain the trauma, but iatrogenic injuries from percutaneous cardiac and vascular procedures, orthopedic procedures, diagnostic kidney and liver biopsies and percutaneous insertion of drainage catheters can result to traumatic arteriovenous fistulas as well. The most common site of iatrogenic injury in the lower extremity is the right common femoral artery [5].

Arteriovenous fistulas of the tibial arteries are a well known complication of fractures, fragment injuries, balloon catheter thrombectomy and atherectomy of the tibial vessels. In our patient, due to the mechanism of the trauma, X-rays were obtained as a first imaging modality and should be the first step to diagnosis in all patients with suspected AVFs for evidence of metal fragments, bullets, abnormal calcifications, and signs of congestive heart failure. The diagnostic algorithm that should be followed requires diagnostic imaging with duplex ultrasound scanning, computed tomographic angiography or magnetic resonance angiography. Due to the mechanism of the trauma and the metal fragment in our patient, after the X-rays and the duplex ultrasound scanning, we performed a catheter-based angiography for definitive treatment. The goals of the treatment were the closure of the fistulous opening, the restoration of normal hemodynamic flow and the exclusion of the traumatic false aneurysm of the anterior tibial artery.

Until recently, open surgery was the only option for treating tibial aneurysms and AVFs if conservative therapy fails. However, due to high morbidity and mortality rates and the advances in endovascular techniques, the use of endovascular treatment is the treatment of choice with high technical success rate [7,8]. In our patient, the endovascular treatment was accomplished by placement of a stent-graft, but transcatheter embolization and combination of these two modalities can be used as well. The endovascular procedure was performed under dual antiplatelet treatment with clopidogrel 75 mg and acetylsalicylic acid 100 mg, which was continued once daily for three months. After this period, the patient continues treatment with clopidogrel 75 mg once daily, as monotherapy.

## Conclusion

Due to the advances in endovascular techniques and the higher morbidity and mortality rates of open repair in treating traumatic aneurysms of the and arteriovenous fistulas of the lower extremity, the use of endovascular treatment can be recommended as the treatment of choice with high technical success rate.

## References

[1] Murakami H, Izawa N, Miyahara S, Kadowaki T, Morimoto N, Morimoto Y (2011). A true aneurysm of posterior tibial artery. Ann Vasc Surg.

[2] Ferrero E, Viazzo A, Robaldo A, Ferri M, Piazza S, Cumbo P (2011). True giant aneurysm of the tibio-peroneal trunk: case report and review of the literature. Vasc Endovascular Surg.

[3] Verbrugghe P, Vandekerkhof J, Baeyens I (2011). Pseudoaneurysm of the anterior tibial artery: a complication of ankle arthroscopy. Acta Chir Belg.

[4] Cronenwett JL, Walsh DB, Garrett HE, Hanover NH, Tenn M (1988). Tibial artery pseudoaneurysms: delayed complication of balloon catheter embolectomy. J Vasc Surg.

[5] Rudström H, Bergqvist D, Ogren M, Björck M (2008). Iatrogenic vascular injuries in Sweden. A nationwide study 1987. -200 Eur J Vasc Endovasc Surg.

[6] Singh D, Ferero A (2013). Traumatic pseudoaneurysm of the posterior tibial artery treated by endovascular coil embolization. Foot Ankle Spec.

[7] Thalhammer C, Kirchherr AS, Uhlich F, Waigand J, Gross M (2000). Postcatheterization pseudoaneurysms and arteriovenous fistulas: repair with percutaneous implantation of endovascular covered stents. Radiology.

[8] Onal B, Kosar S, Gumus T, Ilgit E, Akpek S (2004). Postcatheterization femoral arteriovenous fistulas: endovascular treatment with stent-grafts. Cardiovasc Intervent Radiol.

